# Micro-Nano Bubbles: A New Field of Eco-Friendly Cleaning

**DOI:** 10.3390/nano15070480

**Published:** 2025-03-23

**Authors:** Nan Guan, Yao Wang, Jun Hu, Lijuan Zhang

**Affiliations:** 1 Shanghai Institute of Applied Physics, Chinese Academy of Sciences, Shanghai 201800, China; guannan@sinap.ac.cn (N.G.); wangyao@sinap.ac.cn (Y.W.); 2 University of Chinese Academy and Sciences, Beijing 100049, China; 3 Shanghai Synchrotron Radiation Facility, Shanghai Advanced Research Institute, Chinese Academy of Sciences, Shanghai 201204, China; 4 Institute of Materiobiology, College of Science, Shanghai University, Shanghai 200444, China; 5 Xiangfu Laboratory, Jiashan 314102, China

**Keywords:** micro-nano bubbles, surface cleaning, contaminant removal, eco-friendly technique

## Abstract

Due to increasing public awareness of environmental concerns and stricter cleaning process requirements, traditional cleaning technologies characterized by high pollution, excessive energy consumption, and substantial damage are insufficient to meet contemporary demands. There is an urgent need for efficient, low-damage, and environmentally friendly cleaning technologies. In recent years, the rapid advancement of micro-nano bubbles (MNBs), which exhibit unique physicochemical properties, have emerged as a promising solution for green cleaning applications. This review begins with an overview of the benefits of MNBs in cleaning processes, followed by an in-depth analysis of the factors influencing their cleaning effectiveness as well as the possible mechanisms involved. Additionally, the producing and application of MNBs across various cleaning scenarios are summarized. Finally, prospects for their development are discussed. Research and advancements in MNB preparation technologies are expected to boost their applicability and commercialization in a greater variety of cleaning contexts in the future.

## 1. Introduction

Cleaning is a crucial process not only in industrial applications but also in daily life. It plays a vital role in quality assurance within industrial manufacturing, occurring at several key stages of the process. The quality of product cleaning directly influences the final product yield [1]. Maintaining clean equipment components can reduce fatigue intensity and extend their usage lifetime [2,3]. For medical safety, cleaning is one of the key steps. Medical devices that are reused must be cleaned and disinfected regularly in healthcare settings [4]. If medical instruments are not cleaned properly, this may lead to unnecessary healthcare-associated infections, which can cause morbidity and delay the recovery of patients [5]. Moreover, cleaning represents the last barrier to ensure the safety of food. There is evidence that many foodborne illnesses are directly linked to inadequate surface cleaning and disinfection procedures in food processing environments [6]. A further concern for food safety is pesticide residues [7]. For the food industry to eliminate residual pesticides and microorganisms, effective cleaning procedures are essential. At present, cleaning technology is recognized as a vital component of ensuring industrial precision, protecting human health, and maintaining ecological balance.

Numerous methods have been developed for cleaning purposes, with chemical cleaning being the most widely employed. Chemical reactions are involved in this method to dissolve or transform surface contaminants and it is characterized by its high efficiency and broad applicability. However, it may cause corrosive effects on surfaces and have irreversible environmental consequences [8,9]. In contrast, physical cleaning employs physical methods, such as mechanical forces and various forms of energy (e.g., acoustic, optical, and electrical), to remove surface contaminants. Although environmentally friendly, this approach typically consumes significant energy and surface structures may be damaged by high-intensity forces [10,11,12]. As a primary method of microbial cleaning, enzymes are used to catalyze the transformation of pollutants in a non-toxic and harmless manner [13]. Although this method is considered non-destructive and eco-friendly, it unfortunately exhibits lower efficiency and has a limited range of degradable contaminants.

While traditional cleaning technologies have been crucial in various cleaning scenarios, their inherent limitations have become more apparent when compared with emerging demands for high efficiency, minimal damage, and pollution-free processes. Three main challenges illustrate this particularly well. Firstly, the increasing precision of products necessitates higher levels of cleaning effectiveness. As an essential step in manufacturing, cleaning quality directly affects subsequent processes and product yield [14]. Secondly, the potential for damage from cleaning methods cannot be overlooked. Chemical cleaning techniques pose a risk of corrosion and physical cleaning methods may compromise surface integrity, creating challenges in removing contaminants without damaging surfaces [15]. In addition, public concern over environmental issues has resulted in the development of environmentally friendly cleaning technologies becoming an imperative trend. The misuse of cleaning agents can harm human health and threaten ecological balance [16]. Moreover, the rinse water generated after chemical cleaning is also estimated to amount to billions of tons, raising significant concerns regarding the sustainability of the environment [17].

To address challenges caused by traditional cleaning technologies, there is an urgent need to develop innovative cleaning systems that leverage the synergistic effects of physical and chemical interactions. New opportunities for efficient, non-destructive, and green cleaning have emerged with the development of micro-nano bubble (MNB) technology. MNBs are defined as bubbles with diameters smaller than 100 µm, with microbubbles (MBs) ranging from 1 to 100 µm and nanobubbles (NBs) being less than 1 µm in diameter [18]. MNBs exhibit unique physicochemical properties [19], including high stability, adjustable size, negative surface charge, and the capacity to spontaneously generate free radicals. MNBs are used in this technology for cleaning purposes, and it is usually used alongside physical cleaning methods such as ultrasonic cleaning, high-pressure spraying, and jetting. Numerous studies have been conducted on the use of MNBs in clean technology, as they are capable of performing tasks that conventional cleaning methods are not able to accomplish. Takahashi et al. demonstrated that ozone MNB solutions are efficient at removing highly challenging contaminants such as amorphous carbon layers from high-dose ion-implanted photoresists [20]. MNBs are also remarkably effective in removing biofilms that threaten public health and food safety, even on uneven surfaces and within narrow crevices [21,22].

**Figure 1:**
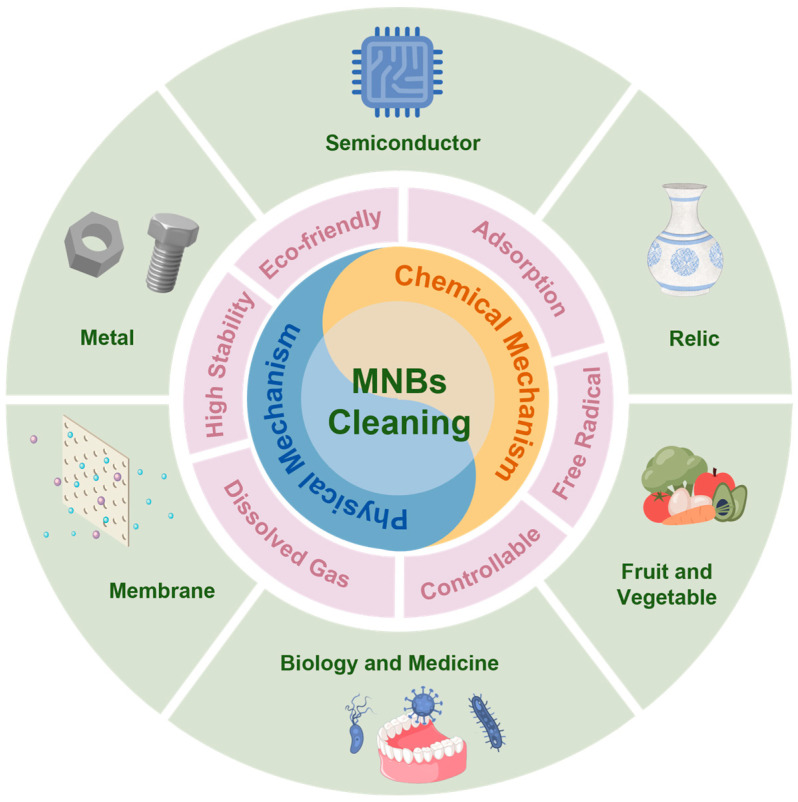
An overview of the properties, mechanisms, and applications of MNBs along the cleaning process.

This review aims to elucidate the application of MNB technology in cleaning processes. The discussion emphasizes the benefits of MNBs for cleaning, reviews the factors influencing the efficacy of this cleaning method, and explores the potential mechanisms involved. A comprehensive overview of the produced methods and applications of MNBs to various cleaning scenarios is also provided in the review. Figure 1 presents all the content involved in this review. Finally, we evaluate the potential of MNBs as an alternative to chemical cleaning agents, with the objective of enhancing cleaning efficiency while minimizing surface damage.

## 2. Properties in Cleaning

MNBs have inherent and unique advantages in cleaning due to their properties, including high stability, adjustability, easy combination with pollutants, high dissolved gas concentration, generation of free radicals, and non-pollution.

### 2.1. High Stability

A bubble’s lifetime is inversely related to its size, according to the classical Epstein–Plesset equation [23], which implies that NBs of the order of hundreds of nanometers have lifetimes measured in tens of microseconds. However, experimental results have proved that NBs in aqueous solutions can persist for several hours or even days [24,25,26]. MNBs in liquids have lower buoyancy than millimeter-diameter bubbles, resulting in a much slower ascent rate [27]. Long-term effects are achieved through this prolonged existence, thus enhancing the effectiveness of cleaning.

### 2.2. High Mass Transfer Efficiency

Compared to traditional aeration methods that produce macro-bubbles, MNBs exhibit distinguishing characteristics, including their small size, large specific surface area, and high internal gas density. Mass transfer rates at gas–liquid interfaces are directly related to the mass transfer area [28]. MNBs have a greater mass transfer area than conventional bubbles, thus increasing mass transfer efficiency. Experimental results from Matsumoto indicates ozone MNBs enhance the mass transfer efficiency of ozone and increase its half-life [29]. Consequently, MNBs possess high mass transfer efficiency, allowing gases to dissolve into liquids [30] and remain in liquid form for a long period of time [31]. This facilitates the dissolution of gases into liquids and allows them to persist in liquids for an extended period. The importance of this is particularly apparent when utilizing MNBs in cleaning applications. Several studies have demonstrated that dissolved gases increase the efficiency of cleaning and also mitigate the erosion caused by cleaning [32,33]. Furthermore, MNBs may also extend the effective duration of gases, especially those that are prone to escaping from a liquid, such as ozone [34].

### 2.3. Adjustability

MNBs can be controlled in terms of size and concentration. Cavitation is a technique for creating MNBs that relies on the principle that a decrease in pressure within a moving fluid induces vaporization, which leads to the formation of bubbles. The pressure and temperature of fluid can be adjusted to alter the size of bubbles produced by hydrodynamic cavitation [35]. Alternatively, the characteristics of MNBs generated through acoustic cavitation are determined by the power, frequency, and duration of the ultrasounds [36,37,38]. As part of pressure variation methods, MNBs are produced by regulating the solubility of gas in solutions, where factors such as water/air saturation pressure, surface tension, and length of depressurization play an important role in determining the concentration of MNBs [39,40]. Additionally, the membrane method for fabricating MNBs involves injecting gas into flowing liquid through the pores of a porous membrane in order to separate liquid from gas. MNBs can be controlled by adjusting the gas pressure and membrane pore size [41]. It is also important to note that the concentration and size of MNBs are also affected by the surrounding liquid environment, including temperature, pH, electrolytes, and the solubility of gases. By controlling the concentration and size of MNBs, targeted cleaning solutions can be implemented in various situations, thus increasing their applicability.

### 2.4. Adsorb Contaminant Easily

Generally, the surface of a MNB is negatively charged and hydrophobic, enhancing the adsorption capacity and facilitating interactions with pollutants, resulting in a more effective cleaning process. MNBs are characterized by an elevated zeta potential resulting from the accumulation of OH- at the bubble interface [42]. Adsorption characteristics at bubble surfaces are largely determined by the zeta potential. There is a markedly superior capacity of metal ions to adsorb in O_2_-MNBs (with an electrical potential of −45 to −34 mV) as compared to air-MNBs (with an electrical potential of −20 to −17 mV) [43]. It is difficult for a macro-bubble to contact contaminants trapped within structures due to capillary action. In contrast, MNBs, since they are smaller, can reach smooth surfaces as well as into narrow gaps and grooves. Furthermore, the nano-scale dimensions of NBs and their ability to reduce liquid surface tension result in a larger specific surface area, which increases the likelihood of combination with contaminants [44].

### 2.5. Generate Free Radicals

During the shrinkage or rupture of MNBs, rapid and intense changes result in ion accumulation at the interface [20], increasing the chemical potential of the interface [45,46]. This phenomenon can spontaneously generate free radicals or other reactive oxygen species (ROS), a crucial chemical mechanism that contributes to the cleaning efficacy of MNBs. In addition, sound, light, or electrical stimuli [47,48] in association with gases (e.g., oxygen and ozone) [20,49,50] are effective for generating MNBs, which in turn help to achieve a superior level of cleaning.

### 2.6. Environment-Friendly

For effective cleaning, traditional cleaning processes frequently rely heavily on chemical cleaning agents. However, the reliance on these agents increases overall cleaning costs. A significant challenge is also presented by managing the waste liquids generated after cleaning. Inadequate treatment of these waste liquids can cause severe environmental pollution and their disposal further complicates both costs and procedures [51]. In contrast, MNBs are regarded as a pollution-free alternative that is both cost-effective and readily available. As MNBs are liquid-based and consist primarily of gas and pure water, they are easily integrated into existing processes. It is also possible to easily remove them after use from a cleaning system without damaging the system or the environment.

## 3. Influencing Factors

To gain a deeper understanding of MNBs’ properties and extrapolate their cleaning mechanisms, it is very necessary to analyze the factors influencing the cleaning efficiency of MNBs. Several studies have shown that the cleaning efficiency of MNBs is primarily influenced by the types of gas, the size of the bubbles, and the methods of cleaning that are used.

### 3.1. Gas Types

The cleaning efficiency of MNBs is influenced by the types of gases used in their generation. Commonly employed gases for producing MNBs in cleaning applications include air, O_2_, N_2_, H_2_, O_3_, and CO_2_. Because of its low cost and availability, air is the most widely used source of gas. The oxidative properties of O_2_ and O_3_ enhance the efficacy of cleaning by causing organic matter to decompose by generating free radicals [52]. As a byproduct of electrolysis, H_2_-MNBs provide superior rectification diffusion rates when compared to other gases, resulting in greater cleaning efficiency than N_2_-MNBs or Ar-MNBs [53]. The high solubility of CO_2_ in water enables the formation of H_2_CO_3_, resulting in a decrease in the pH of the solution and thus improving the cleaning effect when compared with air and N_2_-MNBs [54]. It is noteworthy that a systematic investigation into the comparative cleaning efficiencies of these gases under identical cleaning conditions remains necessary.

### 3.2. Bubble Sizes

MNB sizes are significantly influenced by the methods and parameters used during their formation. Solvent exchange is considered a technical representative for generating interfacial nanobubbles (INBs) for cleaning purposes. As a result of the difference in dissolved gas content before and after the solvent exchange, a local supersaturation is created at the interface which facilitates the nucleation and growth of NBs [55]. There is evidence that the size of the bubbles is affected by factors such as the substrate’s properties, the amount of gas in the solvent, and the rate of exchange [56,57,58]. The pressurized dissolved gas principle is widely used to generate MNBs in cleaning applications. Based on Henry’s Law, this method generates MNBs by dissolving gas in liquid under high pressure, resulting in a supersaturated solution. MNBs are formed after excess gas is precipitated from the solution by reducing the pressure [59]. It is also common to use hydrodynamic cavitation to create MNBs in cleaning operations. Due to high flow rates, a liquid experiencing this phenomenon experiences a sudden drop in pressure, causing the local static pressure to fall below its saturation vapor pressure and causing a phase change. Cavitation bubble nucleation, growth, and rupture are commonly associated with this process. An increase in flow rate is typically achieved by using throttling elements (such as Venturi tubes), vortex-based devices (such as vortex diodes), or mechanical rotating shear devices [60]. MNB sizes are primarily determined by liquid flow rate in this context. Similarly, acoustic cavitation is a technique for generating MNBs via ultrasonically stimulating changes in air pressure with liquid. Bubble sizes may be affected by several parameters, such as ultrasonic frequency, power, and duration [61]. In addition, MNBs produced through electrolysis are also used to clean surfaces. Via electrolysis, water is decomposed, producing O_2_ and H_2_ at the anode and cathode, respectively [62]. The increased supersaturation allows MNBs to grow near the electrodes due to the nucleation of the bubbles. Electrolysis generates MNBs in a variety of sizes, depending on the electrode properties, current density, voltage, and electrolyte concentration [63]. Different methods of measuring bubble sizes provide information at different scales. The measurement of MNB sizes may be performed using atomic force microscopy (AFM), optical microscopy (OM), a high-speed camera (HSC), dynamic light scattering (DLS), nanoparticle tracking analysis (NTA), particle-counting spectrometry (PCS), or sonochemical luminescence (SCL). Table 1 summarizes the advantages and disadvantages of various MNB generation methods, along with the typical size distribution of the bubbles produced.

Efficiencies of cleaning are directly linked to the size of MNBs. MNBs have been shown to provide a significantly greater cleaning effect than millimeter-scale bubbles produced by conventional aeration techniques [78]. There is a negative correlation between the size of MNBs and their cleaning performance [79]. According to Lin et al., MNBs of 3.2 μm in average diameter enhance plaque removal efficacy by 11.1% compared with MNBs of 5.5 μm in average diameter [80]. Li et al. demonstrated that MNBs with a size distribution of 15–135 μm reduce transmembrane pressure by 35% as compared to MNBs with a size distribution between 50–450 μm. It is evident from this finding that smaller-sized MNBs are more promising for controlling membrane fouling [81]. Due to their substantial specific surface area, smaller bubbles are more effective at cleaning, because contaminants are more likely to interact with them. Smaller bubbles are also less susceptible to surface structures, allowing them to penetrate crevices and grooves. Moreover, the high mass transfer efficiency associated with smaller diameters along with the high gas density within MNBs may also contribute to enhanced cleaning performance [34,82].

### 3.3. Cleaning Methods

An important factor affecting the cleaning efficiency of MNBs is the method of cleaning employed. For easily removable contaminants, soaking in a MNB solution can yield optimal cleaning results. Nevertheless, external forces are often required to enhance the cleaning process for more stubborn contaminants. Alternating flow is a commonly used auxiliary method in cleaning involving MNBs [83]. Using this technique increases the likelihood of contaminants interacting with MNBs and effectively dislodges those contaminants from surfaces as well. MNBs’ effects can be amplified by the shear stress exerted on surfaces by liquid flow during spraying [20]. Furthermore, the use of ultrasound may further improve the effectiveness of MNBs in terms of cleaning. Ultrasound at low frequencies induces bubbles to contract or collapse, resulting in microjets that weaken the adhesive forces that bind contaminants to surfaces. It is also believed that the liquid flow and the generation of MNBs triggered by ultrasound contribute positively to cleaning efficiency [84].

Further, the cleaning efficacy of MNBs can also be affected by factors such as temperature [85], pH [86,87], cleaning time [22], and surfactants [88].

## 4. Cleaning Mechanisms

At present, there is no conclusive understanding of the principles underlying MNB cleaning. In this review, various mechanisms identified in previous research are summarized. Based on their different modes of action, these mechanisms can be categorized into physical and chemical mechanisms.

### 4.1. Physical Mechanisms

**Figure 2:**
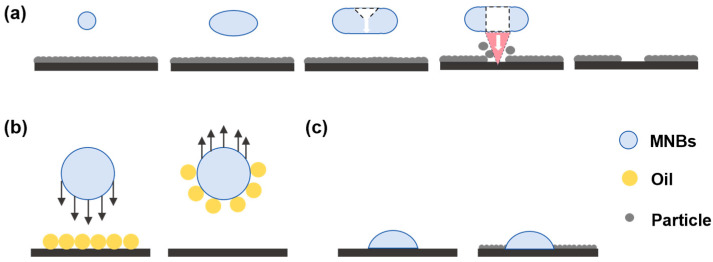
Physical mechanisms of MNBs: (**a**) altering the adhesions, (**b**) departing from surface with contaminations, and (**c**) reducing absorption.

As shown in Figure 2, three primary mechanisms contribute to the improvement of cleaning efficacy by MNBs: weakening the adhesive forces between contaminants and surfaces, facilitating contaminant desorption, and preventing secondary contamination.

The generation of surface NBs alters the interactions between contaminants and surfaces. The increased likelihood of heterogeneous nucleation results in surface NBs preferentially forming around contaminants. By redefining the anchoring forces acting on contaminants, NBs may reduce their adhesion to surfaces, thereby promoting detachment [64]. In addition, the collapse of MNBs near solid surfaces results in liquid jets that decrease the binding force between contaminants and surfaces, allowing them to be separated from one another [89]. It was demonstrated using high-speed cameras for particle tracking that MBs expand and collapse at boundaries, causing radial impacts and the diffusion of jets that effectively remove particles from surfaces [90]. A significant correlation exists between the micro-jets generated by bubble collapse and the removal of surface particles, with a notable consistency observed between bubble rupture locations and areas exhibiting high particle removal rates.

MNBs can also facilitate the desorption of contaminants from surfaces due to their flotation properties. Particle–bubble interactions, such as collision, attachment, and desorption, are governed by the relative size of the particles and bubbles [91]. When the bubble size is reduced, the total surface area increases, enlarging the likelihood of bubbles and contaminants colliding. The charged characteristics of the MNBs’ surfaces promote the adsorption of contaminants. In adsorbing contaminants, MNBs decrease the specific gravity and increase their buoyancy, which makes them easier to detach from solid surfaces [92]. While cleaning contaminated surfaces with oil and salt, microscope observations revealed the aggregation of MNBs with oil droplets, leading to the subsequent rise of these bubbles and improved cleaning efficacy [93]. Flotation results are attributed to hydrophobic interactions between MNBs and oleic acid which accelerate adhesion, while MB self-contraction facilitates the effective separation of oleic acid.

In addition to aiding in contaminant desorption, MNBs may also enhance cleaning efficiency by reducing adhesion. Cleaning efficiency does not always correlate positively with cleaning time during the cleaning process. After a certain period, prolonged cleaning can decrease efficiency due to contaminants’ secondary attachment. An effective way to mitigate this adhesion is through the use of NBs. According to Hu Jun’s research team, pre-generating surface NBs reduces the adsorption of bovine serum albumin by 34% [94]. Based on the research conducted by Fan et al. to prevent membrane fouling, MNBs act as “air bridges” between contaminants and the membrane surface, thus facilitating fouling control by inhibiting adhesion and improving antifouling effectiveness [71].

### 4.2. Chemical Mechanisms

**Figure 3:**
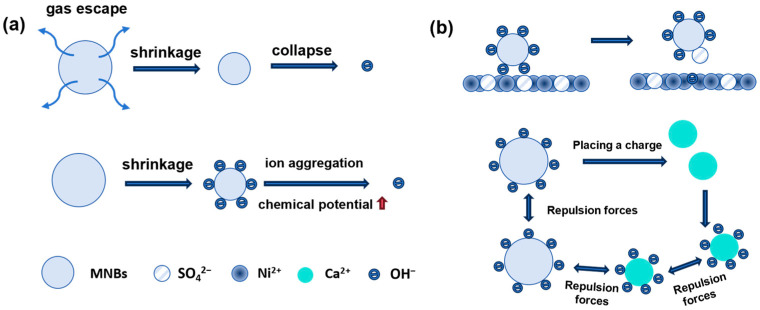
Chemical mechanisms of MNBs: (**a**) reactive ROS and (**b**) ionic interactions.

Figure 3 clarifies the chemical mechanisms underlying the cleaning effectiveness of MNBs that involves reactive ROS and ionic interactions. As MNBs rupture or collapse at the interface [20], they can increase the interfacial chemical potential and lead to the synthesis of hydroxyl radicals (OH) [45,46] and other ROS [9,95]. Researchers have demonstrated that the concentration of OH produced by MNBs is correlated with the concentration of the bubbles using spectrophotometry and electron spin resonance experiments [95,96]. This phenomenon enables MNBs not only to effectively desorb inorganic pollutants and eliminate bacteria and microorganisms but also to oxidize and remove certain organic substances. When sound [20], light [97,98], and an electromagnetic field [99] are present, they enhance the concentration of ROS in solutions containing MNBs, thereby enhancing the effectiveness of cleaning operations. A remarkable increase in the effectiveness of MNB cleaning can be achieved by utilizing oxygen and ozone as gas sources. This improvement can be attributed to the ability of MNBs to enhance gas–liquid contact, prolong the residence time of dissolved gases, and increase mass transfer efficiency during oxidation processes [34,82,100], thus boosting advanced oxidation efficiency.

In addition, ionic interactions represent a vital chemical mechanism through which MNBs can enhance cleaning performance. Takenouchi et al. conducted research showing that H_2_-NBs had a high cleaning efficiency in alkaline electrolytes [101]. H_2_-NBs have been shown to be effective at cleaning and removing sulfate ions from nickel surfaces in their studies. An increased cleaning efficiency is obtained through the exchange of ions between hydroxide ions at the NB interface and sulfate ions remaining on the nickel surface. This facilitates the desorption of sulfate ions from the nickel plating. CaSO_4_ and CaCO_3_ crystals can be inhibited by MNBs through ion interactions [102]. The negatively charged MNBs attract calcium ions to reduce crystal formation. MNBs also impart a surface charge on the crystals, which causes them to remain suspended, thereby decreasing the likelihood of their attachment to membrane surfaces. Moreover, certain gases (for instance, CO_2_ and NH_3_) can alter the pH of the solution when they are dissolved in the liquid, which is intensified by the solubilization effect of MNBs. Contaminants’ dissolving in the presence of H^+^ or OH^−^ are further accelerated.

## 5. Application of MNBs in Cleaning

### 5.1. Semiconductor Cleaning

The cleaning process is an essential component of the semiconductor manufacturing workflow. Statistics indicate that cleaning operations account for approximately 30% of total production time in microelectronics, with associated costs exceeding 20% of the overall budget. There is a direct correlation between the quality of cleaning and the yield rates of subsequent processing stages, as particles larger than half the size of the process nodes can adversely affect the overall fabrication process. The introduction of the 3 nm process node has necessitated even stricter cleaning protocols. A number of other challenges present a significant obstacle to semiconductor cleaning, including the susceptibility of surface structures to damage, the difficulty of cleaning deep trench structures, the removal of photoresists, high chemical reagent consumption, and excessive water usage.

**Figure 4:**
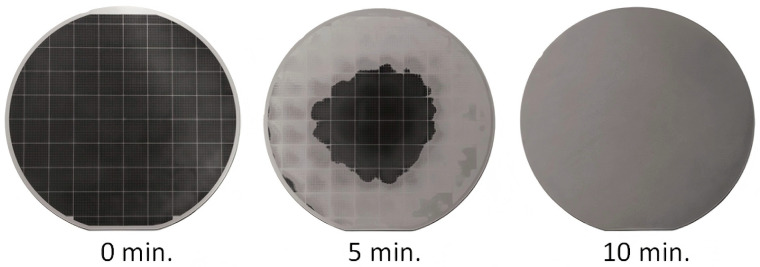
O_3_-NBs remove photoresist [20]. High-dose ion implantation photoresist is shown to be removed from the outer edges of the wafer to the interior at 0, 5, and 10 min after the start of processing. Reprinted with permission from Ref. [20]. Copyright 2012, copyright American Chemical Society.

MNB cleaning technology has shown significant potential for treating nanometer-sized contaminants in semiconductors due to its high removal efficiency, low chemical reagent consumption, and minimal surface damage. Based on molecular dynamics simulations, INBs exhibit a lower jet velocity and a weaker local vorticity, which leads to a smaller amount of cavitation damage to the substrate compared to spherical bubbles [103]. Following the process of alcohol–water exchange, INBs are formed on the surface in the semi-closed environment formed between the O-ring of the AFM liquid cell and the substrate. Researchers have demonstrated that using the alcohol–water replacement method to generate surface INBs effectively eliminates polystyrene nanoparticles from silicon substrates and periodically structured trenches with a 90% removal efficiency without damaging the structure [65]. The growth of INBs at the interface can alter the adhesion forces and facilitate the detachment of nanoparticles from surfaces, whereas their stable properties can also limit the deposition of secondary particles [64]. This approach provides valuable insights for cleaning precision devices, such as micro-electromechanical systems. CeO_2_ is one of the most widely used polishing particles in chemical mechanical polishing (CMP). Research has shown that CO_2_ MNBs reduce the residual CeO_2_ to one-tenth of that in pure water and result in a smoother surface [104]. It shows that CO_2_-MBs have the potential to be an efficient and nondestructive means of cleaning after CMP. Similar results were observed in experiments involving the removal of aluminum oxide nanoparticles from silicon wafer surfaces using MNBs [105]. Clearly, this technology can effectively eliminate nanometer-sized particles without the use of ultrasonics. Moreover, the O_3_-MB immersion cleaning system developed by Yoon et al., which includes cleaning, rinsing, and drying zones, was an efficient method of removing fingerprints and silica particles [106]. Therefore, MNBs have the potential to replace traditional Radio Corporation of America cleaning methods, thereby reducing the reliance on hazardous chemicals. When water containing dissolved ozone passes through a dispersion nozzle, the bubble nuclei generated by turbulence rapidly grow into MBs, which are subsequently released onto the surface of the rotating wafer. The application of MNB solutions of has been shown to improve solubility in water and, by synergizing with OH, increase photoresist removal rates by 30% [20]. Additionally, this technology effectively removes photoresists that have developed amorphous carbon layers due to high-dose ion implantation (Figure 4), which are typically difficult to remove using conventional techniques. A significant technological advance has enabled the efficient oxidative degradation of stubborn contaminants, providing a crucial technological pathway toward improving semiconductor cleaning processes on an environmentally friendly basis.

### 5.2. Membrane Cleaning

It is inevitable that membrane fouling will occur during the membrane separation process. A reduction in membrane flux, diminished separation performance, and a reduced lifespan can result from this phenomenon, ultimately increasing operations costs and adversely affecting the process’s stability and economic viability [107]. Membrane fouling presents a significant barrier to membrane treatment technologies’ advancement, manifesting itself as pore blockage, concentration polarization, and surface deposition. Performing an efficient and low-damage membrane cleaning is imperative for maintaining membrane flux and selectivity as well as extending the service life of the membrane. As physical cleaning methods, air injection, hydrodynamic cleaning techniques, and acoustic cleaning approaches are limited in their efficiency, as well as in the range of contaminants they are able to effectively remove. Although chemical cleaning methods demonstrate satisfactory cleaning efficiency, they may cause damage to the membrane and compromise its inherent selectivity. Studies on membrane fouling have validated the effectiveness of MNBs in mitigating membrane fouling and efficiently removing pollutants from membrane surfaces. MNBs have been applied to nanofiltration (NF) membranes [108], ultrafiltration (UF) membranes [109,110], microfiltration (MF) membranes [111,112], and reverse osmosis (RO) membranes [87,113].

MNBs have been shown to effectively remove surface contaminants without causing membrane damage in recent studies. According to Lee et al., MBs were incorporated into the process of cleaning MF membranes and compared against the effectiveness of various cleaning methods [111]. A contaminated membrane was immersed in a tank of water, where MBs were generated by a bubble generator and introduced into the aqueous phase, thereby facilitating the cleaning of the membrane by the action of the diffuser holes. MBs were found to be more effective than conventional aeration in terms of cleaning. Following treatment with MBs, the infrared peak intensity of contaminants on the MF membrane was significantly reduced and surface roughness was diminished. It has also been shown that MBs can disrupt the structure of the fouling layer on a membrane and modify its morphology, which provides strong evidence that UF membranes are capable of removing surface contaminants efficiently and with little damage. Subsequent studies further demonstrated the versatility of MB cleaning by showing that MBs can enhance the removal rate of particles and colloids from membrane surfaces [114] while maintaining a high level of cleaning efficiency at low concentrations of chemical cleaning agents [73].

Despite the fact that backwashing frequently can help alleviate membrane fouling, these methods typically result in increased energy consumption and shortened membrane lifespans. Enhancing membrane longevity by reducing fouling and maintaining high permeate flux is of paramount importance. By preventing concentration polarization and the deposition of contaminants, MNBs can enhance membrane cleanliness. The cross-flow filtration experiments carried out by Jang et al. evaluated the effects of air MNBs on RO membrane flux, transmembrane pressure, and solute rejection [67]. The researchers developed a crossflow flat rig unit where high-pressure pumping was used to pass a MNB solution through the RO membrane, resulting in in situ membrane cleaning. The results indicated that MNBs could nearly restore the permeate flux of the RO membrane and significantly enhance both the permeate flux and solute rejection. MNBs likely contributed to this improvement by removing the concentration polarization layer, and it is of note that this effect can persist for a considerable period of time. Following studies have reaffirmed the role of MNBs in mitigating surface scaling phenomena [102]. Using air MNBs as a non-chemical anti-scaling agent enhanced the overall performance of membranes and significantly reduced calcium salt precipitation on membrane surfaces, achieving a 30% improvement over commercially available antiscalants. H_2_-MB addition increased membrane water flux and extended membrane service life when treating challenging oilfield brines, thereby improving treatment efficiency when used in extreme environments [115]. With the use of MNBs, the consumption of membranes and operational costs can be reduced, while chemical pollution can be minimized.

### 5.3. Metal Cleaning

The pollutants generated during the processing of metal components primarily consist of machining oils and particles that accumulate on metal surfaces during cutting or stamping operations. As a result of their hydrophobic properties, these oils are difficult to clean with water, presenting the cleaning industry with challenges. The current predominant method for removing oils from metal surfaces is chemical cleaning, supplemented by physical methods such as soaking, ultrasonication, and high-pressure spraying. However, the high energy consumption associated with physical cleaning methods and the misuse of chemical agents result in water waste during rinsing and contribute to environmental degradation. A promising path forward for metal cleaning lies in the use of MNB technology.

**Figure 5:**
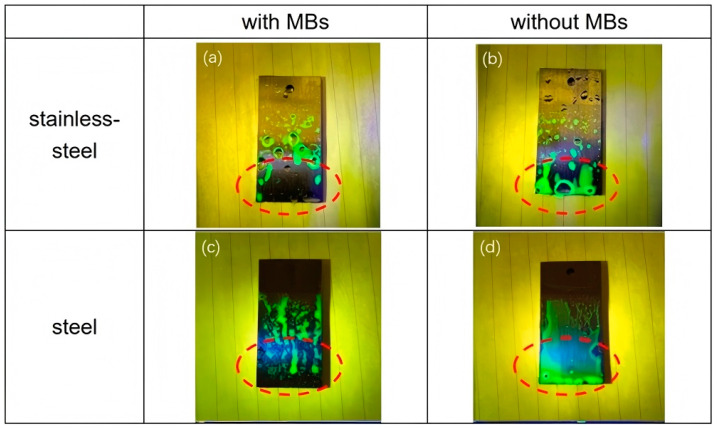
Stainless-steel test pieces after cleaning with (**a**,**c**) or without (**b**,**d**) MNBs [79]. Reprinted with permission from Ref. [79]. Copyright 2022, copyright American Chemical Society.

Minagawa et al. conducted a study utilizing high-speed cameras to determine the effectiveness of MBs generated through pressurized dissolution methods for removing lubricating oil from stainless steel plates when utilizing immersion washing and flowing liquid cleaning methods [116]. The cleaning process was observed to have bubbles penetrate the oil layer, which caused the oil contaminants to detach from the metal surface and rise. The results indicate that MB water significantly improves the cleaning efficiency of lubricating oils, with smaller oil viscosities and smoother surfaces leading to better cleaning results. Additionally, pH levels significantly influence cleaning efficacy; improvements in lubricating oil cleaning efficiency are pronounced in acidic and neutral solutions, while the role of MBs in alkaline solutions appears less significant [117]. An essential part of the effectiveness of oil contaminant cleaning is influenced by the quality of water and the type of gas used, likely as a result of variations in the dynamic behavior of MBs generated by different water and gas types, as well as their correlation to free radicals [118]. As shown in Figure 5, the use of MBs significantly improves the removal of oil from stainless steel surfaces [79]. In addition to enhancing cleaning efficiency, MBs facilitate oil contaminants’ flotation on the surface of clean water. This allows for proper collection and treatment, which significantly reduces emulsified wastewater and promotes water recycling.

### 5.4. Biological and Medicine Cleaning or Sterilization

The significance of preventing non-specific protein adhesion on the surfaces of medical devices is paramount. Various strategies have been employed to develop antifouling surfaces that minimize the adsorption of proteins, cells, and bacteria [119]. Hu et al. examined the effect of NBs on the adhesion and removal of bovine serum albumin (BSA) from hydrophilic [94] and hydrophobic surfaces [120]. A marked reduction in BSA adsorption on surfaces treated with NBs was observed in their study. Additionally, the electrochemical treatment of surfaces coated with BSA produced NBs that removed approximately 20% of the adhered BSA. As the number of treatments increased, cleaning efficiency improved further. In conclusion, these results suggest that NBs could be a cost-effective and detergent-free method for preventing contamination and cleaning surfaces that have been contaminated.

**Figure 6:**
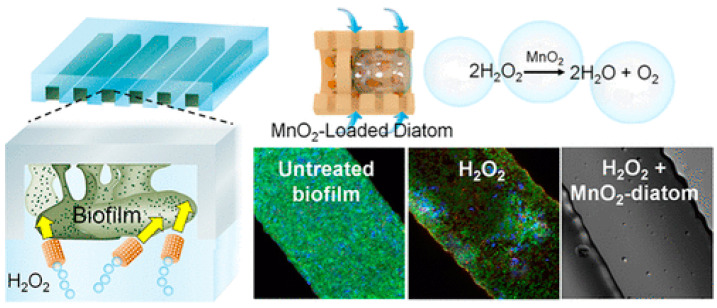
Diatom nanosheets doped with MnO_2_ produce O_2_-MBs that destroy the biofilm. A number of bacteria are killed after H_2_O_2_ molecules diffuse into the biofilm [77]. Reprinted with permission from Ref. [77]. Copyright 2018, copyright American Chemical Society.

There is a serious threat to human health posed by bacterial biofilms that form on the surfaces of various living tissues, medical devices, and engineering materials. Biofilm removal remains a challenging task in spite of extensive research efforts. Reactive materials that generate MBs may prove useful in cleaning surfaces that are biofouled [77]. Microstructures composed of manganese dioxide nanosheets and diatomaceous earth can continuously release oxygen and generate MBs as shown in Figure 6. MNBs coalesce and convert surface energy into mechanical forces that disrupt the biofilm matrix, letting H_2_O_2_ molecules diffuse into the biofilm and kill 99.9% of bacteria. This presents a promising new avenue for the effective development of active antibacterial membrane systems in biomedical and environmental applications.

For maintaining oral hygiene, tooth brushing is the most widely used method. For patients with severe gingivitis or periodontitis, toothbrushing may not be sufficient to eliminate all bacteria from the oral cavity, which can lead to gingival bleeding and complicate the process of healing. Research indicates that MNBs have the ability to reduce the bacterial load in the periodontal vicinity without disrupting the balance of the oral microbiome [121]. Lin et al. conducted experiments with a MNB generator operating at varying rotational speeds and sizes to produce NB solutions for cleaning dental plaque on tooth models [122]. MNB water flow was found to be effective at removing dental plaque significantly. Under optimal conditions (nozzle diameter of 0.8 mm and speed of 3527 rpm), the plaque removal efficiency reached 98%. The researchers also found that the size of MNBs affected removal efficiency; smaller bubble diameters resulted in better cleaning efficiency. During orthodontic treatment, MNBs are remarkably effective at removing dental plaque. Using high concentrations of MNBs resulted in a three-fold greater plaque removal rate when compared with distilled water, according to Sueishi et al. Thus, MNBs are expected to reduce the incidence of dental caries during orthodontic treatment [21].

NB cleaning has also demonstrated remarkable effects on alleviating skin inflammation. H_2_-NB baths generated through electrolysis have been shown to increase serum antioxidant capacities and reduce levels of the inflammatory marker C-reactive protein in patients [123]. This effect has been validated in both healthy volunteers and patients with collagen diseases and dermatomyositis. As well as enhancing the appearance of patients’ skin and alleviating inflammation symptoms, H_2_-NB baths exhibit short-term antioxidant and anti-inflammatory effects. They provide long-term and mid-term health benefits as well, potentially contributing to the maintenance of overall health and reducing infection mortality. Furthermore, NB water baths have shown remarkable effectiveness in treating Harlequin Ichthyosis (HI). With O_2_-NBs, deeper penetration is achieved and oxygen is delivered with greater efficiency, alleviating the hypoxic conditions that are caused by the thickening and cracking of the skin associated with HI. O_2_-NBs promote tissue repair and mitigate the severity of symptoms such as skin fissures and desquamation, potentially reducing the risk of infection in HI patients and leading to significant improvements in overall skin health [124].

### 5.5. Fruit and Vegetable Cleaning

Pesticide residues and microorganisms pose concerns regarding the safety of fruit and vegetable consumption that warrant serious attention. In most cases, fruit and vegetables are cleaned by hydraulic rinsing, often supplemented by physical assistance and ultrasonic cleaning. Produce can easily be damaged by hydraulic rinsing and the process is generally recommended for thicker-skinned fruits and vegetables. In contrast, ultrasonic cleaning can generate strong vibrations and elevated water temperatures, potentially rupturing plant cells and leading to the loss of nutritional components.

Initially, air MB immersion has yielded some success in cleaning impurities from blueberries, tomatoes, and spinach [54]. However, the effectiveness of this method to remove pesticide residues and inactivate bacteria was limited. The use of MB-assisted disinfectants has demonstrated a significant enhancement in antibacterial activity against pathogens such as Escherichia coli and Listeria monocytogenes, with carbon dioxide MBs showing the greatest efficacy. Ushida’s research has indicated that MNB solutions utilized under alternating flow conditions markedly improve cleaning efficacy, reducing in bacterial counts by an order of magnitude compared to rinsing with deionized water alone [83]. Ozone is one of the most effective oxidizing agents for sterilizing and disinfecting food [125]. However, its high instability in water restricts its practical applications. Due to their enhanced stability and mass transfer efficiency, MNBs may alleviate this limitation. Ozone has been proposed as a gas source for MNBs to remove pesticide residues and bacteria from fruit and vegetable surfaces [126]. The generation of OH by ozone MNBs may enhance the oxidative capacity of ozone, as well as the ability to remove pesticide residues and bacteria more effectively. In comparison with those washed with municipal water, the residual concentrations of chlorantraniliprole and dimethomorph after OMB treatment were determined to be 16.1% and 19.4%, respectively. Meanwhile, the levels of S. Typhimurium on cabbage leaves decreased by 2.9 log CFU g^−1^, while E. coli decreased by 2.9 log CFU g^−1^, and the bacterial populations fell below the detection limit (1 CFU 100 mL^−1^) [127]. Using this method prevents additional damage to the leaves, ensuring the quality of the vegetables. A notable difference between O_3_-MNB solutions and ozone millimeter bubble solutions is that MNB solutions have superior removal rates for fenitrothion and shorter cleaning durations [78]. MNBs outperformed commonly used chlorine-based chemical cleaners, particularly in the area of cleaning leafy vegetables.

### 5.6. Cultural Relic Cleaning

**Figure 7:**
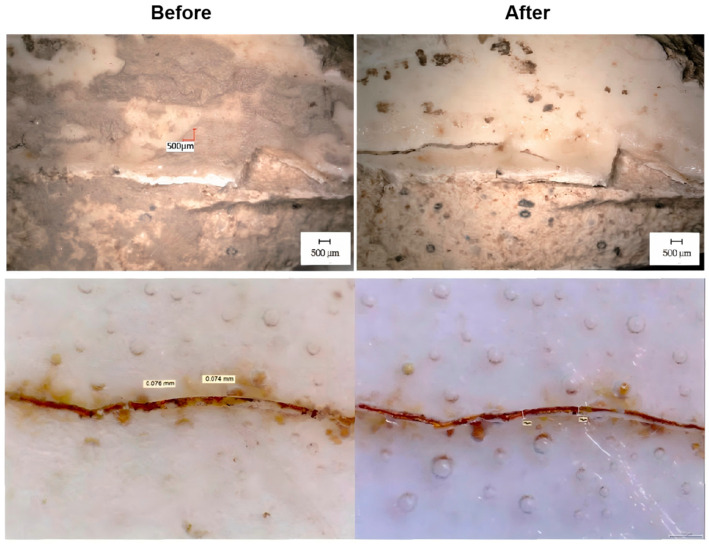
MNBs are used to clean porcelain artifacts [128,129]. Impurities are reduced and cracked surfaces are not deepened after cleaning. Adapted with permission from Refs. [128,129]. Copyright 2017 and 2020, copyright Sciences of Conservation and Archaeology.

Cultural relics represent humanity’s invaluable historical and cultural heritage, with ceramic artifacts forming a significant component of this wealth. Artifacts that have been passed down typically originate from archaeological excavations and are often more complete, requiring only the cleaning of soil stains to restore their original appearance. In contrast, artifacts unearthed from excavation sites and ceramics found through underwater archaeology frequently undergo prolonged weathering or erosion. As a result, glazes may display cracks and contain layers of mineral, organic, and metal oxide contamination. Traditional physical and chemical cleaning methods face distinct technical limitations when it comes to cleaning the surfaces of artifacts. Physical cleaning often necessitates the application of mechanical force to achieve desired results, which can exacerbate existing cracks and damage the luster of the glaze. Similarly, chemical cleaning methods may corrode fragile glazes, interfering with the artifacts and hindering the maximal retention of information. MNB technology has been employed to remove contaminants from the surfaces of ceramic artifacts and has been practically tested on porcelain shards excavated from the “Nanhai I” shipwreck [128] and the Qinglong Town ruins [129] shown in Figure 7. Researchers evaluated the effects of MNB cleaning, ultrasonic cleaning, and steam cleaning on ceramic shards in terms of color difference, glossiness, glaze condition, and crack status. By utilizing MNB cleaning, the removal rate of contaminants was significantly enhanced without damaging the glaze, which validates the effectiveness of this technology for cleaning ceramics.

Textile relics also constitute a vital part of cultural heritage, and their water-washing treatment represents the most critical pre-treatment step in their restoration, display, and storage processes. The process of cleaning textile relics has been the subject of controversial research for many years. Taking inspiration from the effective and non-destructive cleaning of textiles by MNBs [88,130,131], MNBs are being considered to resolve this thorny issue. According to a multidimensional comparison of different cleaning methods, the combination of gentle brushing with MNB cleaning has demonstrated superior cleaning efficacy and minimized damage [132]. The method can be applied to cotton, linen, and wool textile relics contaminated with organic/inorganic composite stains. It is promising for practical applications in the cleaning of textile relics and offers valuable insights for extending the preservation lifespan of such items.

## 6. Conclusions and Outlook

By providing unique advantages, MNB cleaning technology is emerging as an effective, non-destructive, and environmentally friendly cleaning method with wide-ranging applications. In industrial, medical, and everyday cleaning applications, this technology has demonstrated significant cleaning efficacy that is superior to traditional methods. Various industries, including the cleaning industry, have demonstrated a remarkable economic benefit from MNB technology [132,133,134]. MNBs offer a significant advantage due to their low energy consumption, indicating that the process is energy-efficient [118]. The use of MNBs not only reduces expenditures associated with chemical cleaning agents but also minimizes the costs related to wastewater after cleaning. However, MNB cleaning technology faces numerous opportunities and challenges. Firstly, MNBs can be controlled in terms of size and concentration to provide targeted cleaning solutions in a variety of contexts. Despite the fact that it is crucial to improve cleaning efficacy, there is a dearth of research in this area. Industrial-scale applications of MNB solutions are hindered by the lack of efficient large-scale production of high-purity solutions. Secondly, MNB cleaning technology is predominantly utilized under ambient temperature and pressure conditions, whereas many industrial operations involve extreme conditions (high temperature or pressure), making the application of this technology in such scenarios largely uncharted. Furthermore, the limitations of equipment make in situ observation of NB cleaning difficult. It is also insufficient to conduct simulations in this area. There remains a lack of understanding of the mechanisms through which MNBs enhance cleaning efficiency. Additionally, integrating MNB cleaning technology with other emerging technologies, such as plasma, is anticipated to have synergistic effects [135,136]. However, incorporating plasma, ultrasound, flow, and spraying techniques complicates the dynamics of MNB movement, posing additional research challenges. In summary, MNBs are expected to play an increasingly important role in the cleaning industry with further research.

## Figures and Tables

**Table 1:** Advantages and disadvantages of different MNBs generation methods and the typical size range of MNBs.

MNBs Generation Methods	Advantages	Disadvantages	Size (μm)	Detections	References
Solvent exchange	Good repeatability In situ observation Low energy consumption	Difficult to mass produce	0.02–200 (Air)	AFM	[64,65]
Pressurized dissolved gas	Mature technology Straightforward processes High flexibility	Low efficiency High energy consumption	1–60 (O_3_)	PCS	[20]
			14–56 (Air)	OM	[66]
			0.15–0.25 (Air)	DLS	[67]
			0.02–0.4 (Air)	NTA	[68]
Hydrodynamic cavitation	Enables mass preparation Low energy consumption	Limited uniformity of size Clogged and corroded	10–100 (Air)	HSC	[69]
			0.02–2 (Air)	DLS	[70]
			0.2–0.9 (Air)	NTA	[71]
Ultrasonic cavitation	Controllable bubble size	Low density of bubbles results in limited scalability	100–300 (O_2_)	HSC	[33]
			1–4.5 (Air)	SCL	[72]
			0.05–0.15 (Air)	NTA	[37]
Porous-membrane	Controllable bubble size	Producing porous microstructures faces challenges	10–200 (Air)	HSC	[73]
Electrolysis	Controllable bubble size High gas purity	High consumption Low bubble yield Singular gas species	20–200 (H_2_, O_2_)	HSC	[74]
			10–100 (H_2_, O_2_)	OM	[75]
			0.02–0.2 (H_2_, O_2_)	NTA	[76]
Chemical reaction	High efficiency Low energy consumption	Bubble size control difficult	12 (O_2_)	OM	[77]

## Data Availability

Not applicable.
